# Gut microbiota modulates bleomycin-induced acute lung injury response in mice

**DOI:** 10.1186/s12931-022-02264-7

**Published:** 2022-12-10

**Authors:** Young me Yoon, Cara L. Hrusch, Na Fei, Gabriel M. Barrón, Kathleen A. M. Mills, Maile K. Hollinger, Tania E. Velez, Vanessa A. Leone, Eugene B. Chang, Anne I. Sperling

**Affiliations:** 1 Committee on Immunology, University of Chicago, Chicago, IL USA; 2 Section of Pulmonary and Critical Care Medicine, Department of Medicine, University of Chicago, Chicago, IL USA; 3 Section of Gastroenterology, Department of Medicine, University of Chicago, Chicago, IL USA; 4 Division of Pulmonary and Critical Care Medicine, Department of Medicine, University of Virginia, Box 800546, Charlottesville, VA 22908-0546 USA

## Abstract

**Background:**

Airway instillation of bleomycin (BLM) in mice is a widely used, yet challenging, model for acute lung injury (ALI) with high variability in treatment scheme and animal outcomes among investigators. Whether the gut microbiota plays any role in the outcome of BLM-induced lung injury is currently unknown.

**Methods:**

Intratracheal instillation of BLM into C57BL/6 mice was performed. Fecal microbiomes were analyzed by 16s rRNA amplicon and metagenomic sequencing. Germ-free mice conventionalization and fecal microbiota transfer between SPF mice were performed to determine dominant commensal species that are associated with more severe BLM response. Further, lungs and gut draining lymph nodes of the mice were analyzed by flow cytometry to define immunophenotypes associated with the BLM-sensitive microbiome.

**Results:**

Mice from two SPF barrier facilities at the University of Chicago exhibited significantly different mortality and weight loss during BLM-induced lung injury. Conventionalizing germ-free mice with SPF microbiota from two different housing facilities recapitulated the respective donors’ response to BLM. Fecal microbiota transfer from the facility where the mice had worse mortality into the mice in the facility with more survival rendered recipient mice more susceptible to BLM-induced weight loss in a dominant negative manner. BLM-sensitive phenotype was associated with the presence of *Helicobacter* and *Desulfovibrio* in the gut, decreased Th17-neutrophil axis during steady state, and augmented lung neutrophil accumulation during the acute phase of the injury response.

**Conclusion:**

The composition of gut microbiota has significant impact on BLM-induced wasting and death suggesting a role of the lung-gut axis in lung injury.

**Supplementary Information:**

The online version contains supplementary material available at 10.1186/s12931-022-02264-7.

## Background

Increasing evidence based on both clinical and experimental studies suggest that the microbiome plays an essential role in inflammatory pulmonary conditions such as acute lung injury (ALI) and acute respiratory distress syndrome (ARDS). Characterized by acute and diffuse lung inflammation, ARDS development is most often associated with respiratory infection induced-pneumonia, non-pulmonary sepsis, aspiration of gastric and/or esophageal contents, and major trauma [1–3]. In recent studies, the enrichment of specific commensal species in the lung microbiome community was shown to be closely linked to development and outcome of ARDS among critically ill patients [4–6]. Enteric bacteria *Enterobacteriaceae* were found in lungs of ARDS patients even in the absence of systemic bacterial infection, suggesting that translocation of commensal bacteria from gut to lung can alter the lung microbiota and affect lung health [4, 7]. Furthermore, anaerobic gut commensals including *Bacteroides* and *Enterococcus faecalis* were abundantly found in lungs and correlated with survival in an experimental sepsis model [4]. Therefore, understanding the effect of microbiome on lung injury and inflammation could lead to improved prediction and treatment strategies for ARDS patients.

Intratracheal instillation of bleomycin (BLM) in mice is a commonly used model of lung injury and fibrosis. Unlike systemic bacterial infection, BLM model induces sterile injury without the use of any infectious agent or bacterial components. BLM is an anticancer antibiotic, which was originally isolated from the actinomycete *Streptomyces verticillus* [8]*.* It induces cell death by generating reactive oxygen species and double-stranded DNA breaks, which primarily leads to epithelial injury [8–10]. The response to intratracheal administration of BLM in animals is characterized by two phases, acute and fibrosis. Neutrophilic alveolitis and increased lung permeability take place during the first 7 to 11 days of the acute phase after injury, followed by a fibrotic phase with the contraction of inflammation [8, 11, 12]. Even though the BLM model has been refined over the years, the BLM dosage used to induce pathology in animals varies widely among investigators [13]. The need for a varying amount of BLM even for the same strain of mice suggests that some environmental factors, such as diet and microbiome, may contribute to the susceptibility to BLM-induced lung injury.

Previous studies showed that germ-free animals are protected from BLM-induced mortality, suggesting that the microbiome plays a pivotal role in the BLM model [6, 14]. However, in the context of BLM challenge, the effect of the spontaneous difference in the composition of commensal microbes in specific pathogen-free (SPF) environments remains unknown. Herein, we investigated the hypothesis that spontaneous differences in gut microbial communities between genetically similar animals could contribute to variability in response to BLM challenge. Our findings suggest that the presence of specific gut commensal microbes may be a risk factor for having more severe inflammatory lung diseases and additionally highlight the need for defining microbial environments in laboratories for improving the reproducibility of experimental lung injury studies.

## Materials and methods

### Animal and husbandry

C57BL/6 mice were bred and maintained in two designated animal housing facilities at the University of Chicago. Both facilities were maintained at SPF barrier I level with the use of positively pressurized and individually ventilated caging with automated reverse osmosis watering system. Caging and bedding were autoclaved and changed in biological safety cabinets. Facility A mice were provided with NIH-31 Modified Open Formula (7913, Harlan-Envigo, Indianapolis, IN), and Facility B mice were provided with Teklad Global 18% Protein Rodent Diet (2918, Harlan-Envigo, Indianapolis, IN). Male age-matched germ-free C57BL/6 recipient mice were bred in the University of Chicago Gnotobiotic Research Animal Facility. All animal studies were performed in agreement with the approved IACUC Animal Care and Use Protocol.

### Bleomycin model

Bleomycin for Injection USP (Teva Pharmaceuticals USA, Sellersville, PA) was reconstituted at 3 U/mL in endotoxin-free PBS and stored at − 80 °C until use. Mice were anesthetized with ketamine and xylazine and intratracheally administered 1 U/kg bleomycin in 50 µL volume. Mice were weighed daily and euthanized if weight loss surpassed 25% of the original weight or if their wellness scores dropped.

### Fecal microbiota transfer (FMT)

Conventionalization of germ-free mice in SPF facilities was done by adding dirty bedding and feces from neighboring mouse cages in the respective SPF facilities, twice a week. For FMT between SPF mice, fresh fecal pellets from donor mice were suspended in 1 mL of PBS per pellet (each fecal pellet weighed 60–70 mg), and 0.2 mL of fecal slurry from each donor was combined and passed through an 18G needle 10 times. Mice received 0.2 mL of pooled fecal slurry via oral gavage, three times a week.

### Cellular analysis and flow cytometry

Perfused mouse lungs were dissociated by mincing followed by digestion with 150 U/mL Collagenase D (Gibco, Waltham, MA) and 0.02 mg/mL DNase I (Worthington, Lakewood, NJ) in 10 mL of DMEM plus 5% FCS (X&Y Cell Culture, Kansas City, MO) for 1.5 h. Samples were then treated with ACK lysis buffer to remove residual red blood cells. For flow cytometry, 0.5–1 × 10^6^ cells were suspended in 50 μL of FACS buffer, blocked using 2.4G2 hybridoma supernatants (anti-CD16/32), and then stained with surface antibodies for 30 min at 4 °C. For all intracellular staining, cells were fixed and permeabilized using the Foxp3/Transcription Factor Staining Buffer Set (eBioscience, Waltham, MA), and then stained with intracellular antibodies in perm buffer overnight at 4 °C. Antibody-stained cells were analyzed on an LSRFortessa (BD, Franklin Lakes, NJ) or Aurora (Cytek, Fremont, CA), and data analysis was performed using FlowJo (BD, Franklin Lakes, NJ). Antibodies and dilution factors used for staining are listed in Additional file 1.

### Metagenomics

Metagenomics sequencing analysis of fecal samples from unperturbed SPF mice were performed by Transnetyx (Cordova, TN). Fresh mouse fecal samples were placed in barcoded sample collection tubes containing DNA stabilization buffer and shipped to Transnetyx where DNA extraction, library preparation, sequencing, and the initial analysis were performed. Briefly, genomic DNA was extracted using DNeasy 96 PowerSoil Pro QIAcube HT extraction kit (Qiagen, Germantown, MD) and was converted into sequencing libraries using the KAPA HyperPlus library kit (Roche, Basel, Switzerland). Unique dual indexed adapters were used to ensure that reads and/or organisms were correctly assigned. After quality control, the libraries were sequenced on Illumina NovaSeq platform (Illumina, San Diego, CA) using the shotgun sequencing method (a depth of 2 million 2 × 150 bp read pairs), which enables species and strain level taxonomic resolution. Raw data files were uploaded onto One Codex analysis software and analyzed against the One Codex database consisting of > 115K whole microbial reference genomes, assembled from both of public and private sources. Sequence alignment and taxonomy classification were achieved using the One Codex analysis software through the following three steps. First, every individual sequence (NGS read or contig) was compared against the One Codex database by exact alignment using k-mers where k = 31 [15–17]. Second, based on the relative frequency of unique k-mers in the sample, sequencing artifacts were filtered out of the sample. Third, the relative abundance of each microbial species was estimated based on the depth and coverage of sequencing across every available reference genome.

### 16S rRNA gene sequencing

Sequencing of fecal samples from fecal microbiota transfer experiments was performed by the DFI Microbiome Metagenomics Platform at the University of Chicago. DNA was extracted from freshly frozen fecal pellets using the QIAamp PowerFecal Pro DNA Kit (Qiagen, Germantown, MD), and the V4–V5 region of the 16S rRNA genes were PCR amplified using barcoded dual-index primers. Illumina compatible libraries were generated using the QIASeq 1-step amplicon kit (Qiagen, Germantown, MD), and sequencing was performed on the Illumina MiSeq platform (Illumina, San Diego, CA) in the Functional Genomics Facility at University of Chicago using 2 × 250 paired end reads, generating 5000–10,000 reads per sample. Raw V4–V5 16S rRNA gene sequence data was demultiplexed and processed through the dada2 pipeline [18] into Amplicon Sequence Variants (ASVs). ASVs were identified with the Bayesian RDP classifier up to the genus level and were BLASTed against RefSeq for species-level identification.

Alpha and beta-diversity analyses were performed in R using the *phyloseq* package [19]. Alpha diversity was calculated by Shannon’s diversity index [20]. Principal coordinate analysis (PCoA) was performed based on weighted UniFrac distances [21], and permutational multivariate analysis of variance [PERMANOVA, R function adonis (vegan, 999 permutations)] was used to analyze paired statistical differences in beta diversity [22]. Significantly different taxa were determined using STAMP platform [23]. Benjamini–Hochberg false discovery rate correction was used to correct for multiple hypothesis testing [24].

### Availability of data and materials

All 16s rRNA amplicon and metagenomics sequencing data files are available in the NCBI Sequence Read Archive (Accession: PRJNA903920).

## Results

### C57BL/6 mice housed in two SPF animal facilities respond differently to BLM-induced lung injury

**Fig. 1:**
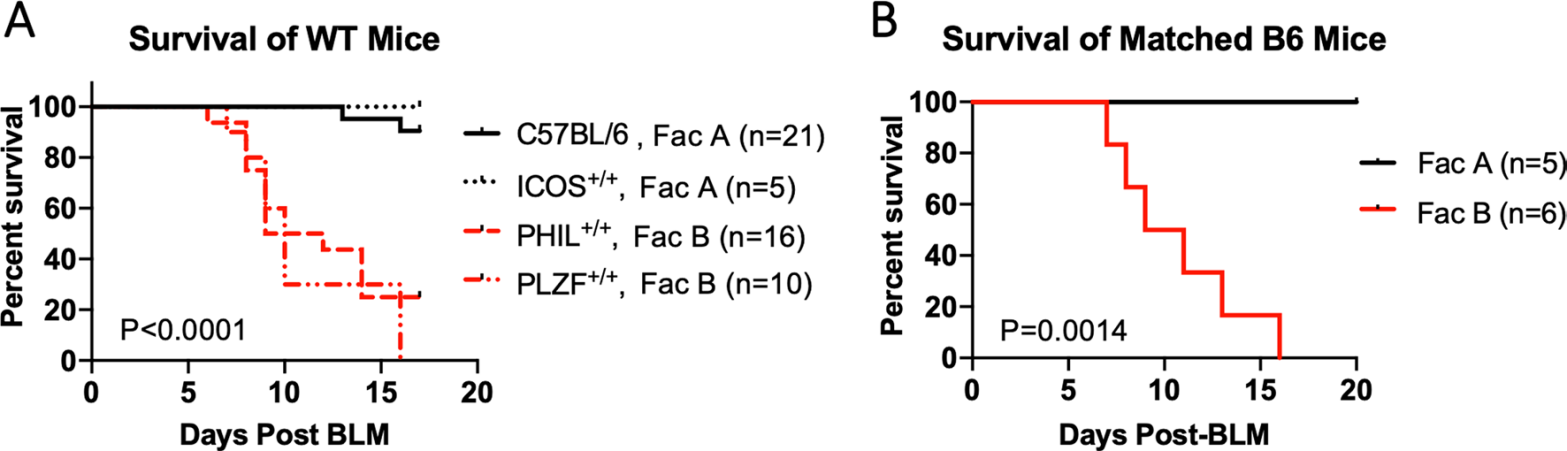
WT mice housed in two SPF housing facilities exhibit differential survival in response to lung injury. The response to bleomycin was measured in WT mice that are all on a C57BL/6 background but born and raised in separate SPF animal housing facilities at the University of Chicago. Mice received 1.0 U/kg bleomycin intratracheally and were monitored daily for weight loss and survival. Mice were euthanized if they lost 25% or more of the original weight. **A** Survival data compiled from multiple experiments. **B** Survival of age matched male B6 mice in two facilities after being challenged with the same stock of BLM on the same day. P-values were obtained from log-rank test

Previous work from our group has shown that intratracheal administration of BLM induces lung injury, including weight loss, lung edema, and neutrophilia, in C57BL/6 mice [12]. We found that C57BL/6 mice and wild-type (WT) littermates of the B6.ICOS^−/−^ strain survived BLM challenge at a dose of 1 U/kg when the experiments were performed in one of our University of Chicago animal housing facilities (Facility A). However, WT littermates from multiple strains on a C57BL/6 background (B6.PHIL and B6.PLZF^−/−^ strains) died at a significantly higher rate when we performed experiments in a different housing facility (Facility B) (Fig. 1A). Both facilities are maintained at the SPF Barrier I level by the same Animal Resource Center of the University. To ensure the exclusion of certain pathogens, these facilities are equipped with directional airflow and provided with irradiated diets and autoclaved caging and bedding. Since both facilities are at the same barrier level, the transfer of animals between the two facilities are allowed, and researchers can enter these facilities in any sequence. Therefore, it was surprising to observe vastly different survival in animals between the facilities. The different colonies of WT littermates on the C57BL/6 background were all born and raised in the respective rooms where the animals were BLM challenged and subsequently monitored. The BLM model is sensitive to the sex and age of the mice and the source of BLM [25, 26]. For example, male mice are significantly more susceptible to weight loss, pro-inflammatory response, and fibrosis in the BLM model [25, 27]. Consistent with this known phenomenon, the facility-dependent difference in BLM susceptibility was prominent in adult male mice while female mice rarely died in both facilities during the present study. Next, we used the same BLM stock and personnel to challenge age- and sex-matched male C57BL/6 mice in both facilities on the same day. Again, we observed a striking difference in survival among matched mice between the two facilities (Fig. 1B). Furthermore, the differential susceptibility to lung injury between mice in the two facilities was observed in a second lung injury model using LPS (Additional file 1: Fig. S1). These data suggest that the phenomenon we observed is relevant for some common pathways induced during lung injury.

### Conventionalizing germ-free mice recapitulates the respective donors’ responses to BLM challenge

We hypothesized that the difference in microbiota in our two animal housing facilities could be responsible for the difference in susceptibility to the BLM challenge. To test this hypothesis, germ-free (GF) C57BL/6 male littermates were conventionalized in either of the two facilities by adding dirty bedding and feces from neighboring cages housing WT SPF mice in respective facilities twice a week. The mice were conventionalized for 2 weeks according to a published study design that demonstrated that the horizontal transfer of bacteria and differentiation of host immune cells are achieved after 2 weeks of cohousing of animals [28].

**Fig. 2:**
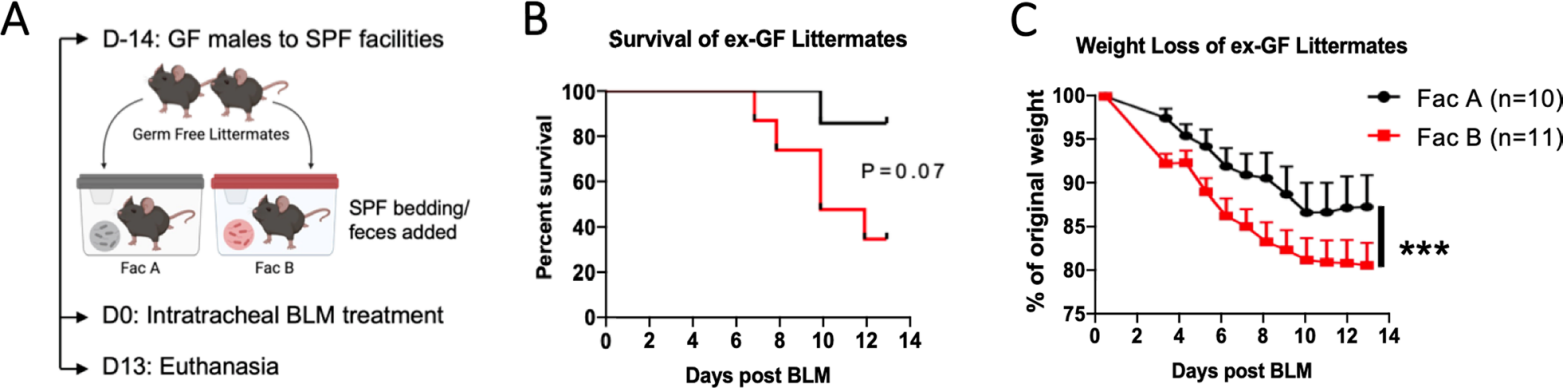
Conventionalizing germ-free littermates in two SPF facilities modulate the severity of lung injury response. **A** Germ-free C57BL/6 male littermates were transferred into either of the two SPF facilities and conventionalized for 14 days by adding dirty bedding and feces from neighboring cages twice a week. Conventionalized ex-GF mice were treated with 1.0 U/kg intratracheal bleomycin and monitored daily for survival and weight loss. **B** Survival and **C** weight loss of conventionalized ex-GF mice after bleomycin challenge. P-values were obtained from log-rank test and 2-way ANOVA, respectively, and depicted as P < 0.05 (*), P < 0.01 (**), P < 0.001 (***)

The conventionalized ex-GF mice were challenged with BLM (Fig. 2A). We monitored body weight as an indicator for the degree of lung injury since there is a significant correlation between body weight loss and lung injury for the BLM model [29]. Mice that lost 25% or more of their starting weight were euthanized even if the mice had sufficiently high wellness scores. Similar to the response of SPF mice, only one of the ex-GF mice in Facility A succumbed to BLM-induced lung injury (10% mortality), while 5 out of 11 (45%) succumbed in Facility B (Fig. 2B). Furthermore, the mice had already lost significantly more weight in Facility B by day 3, suggesting that the early response to lung injury was affected. Weight loss was significantly different throughout the experiment (Fig. 2C). A limitation of our findings in Fig. 1 was that while all the mice were of the C57BL/6 background, the origin of each strain was unknown, thus there could have been some genetic differences in mice. However, these results from conventionalized ex-GF C57BL/6 mice suggest that the weight loss during BLM-induced lung injury is influenced by the microbiota, independent of genetics of the mice.

### Conventionalized ex-GF mice in two SPF housing facilities harbor unique fecal microbiomes

**Fig. 3:**
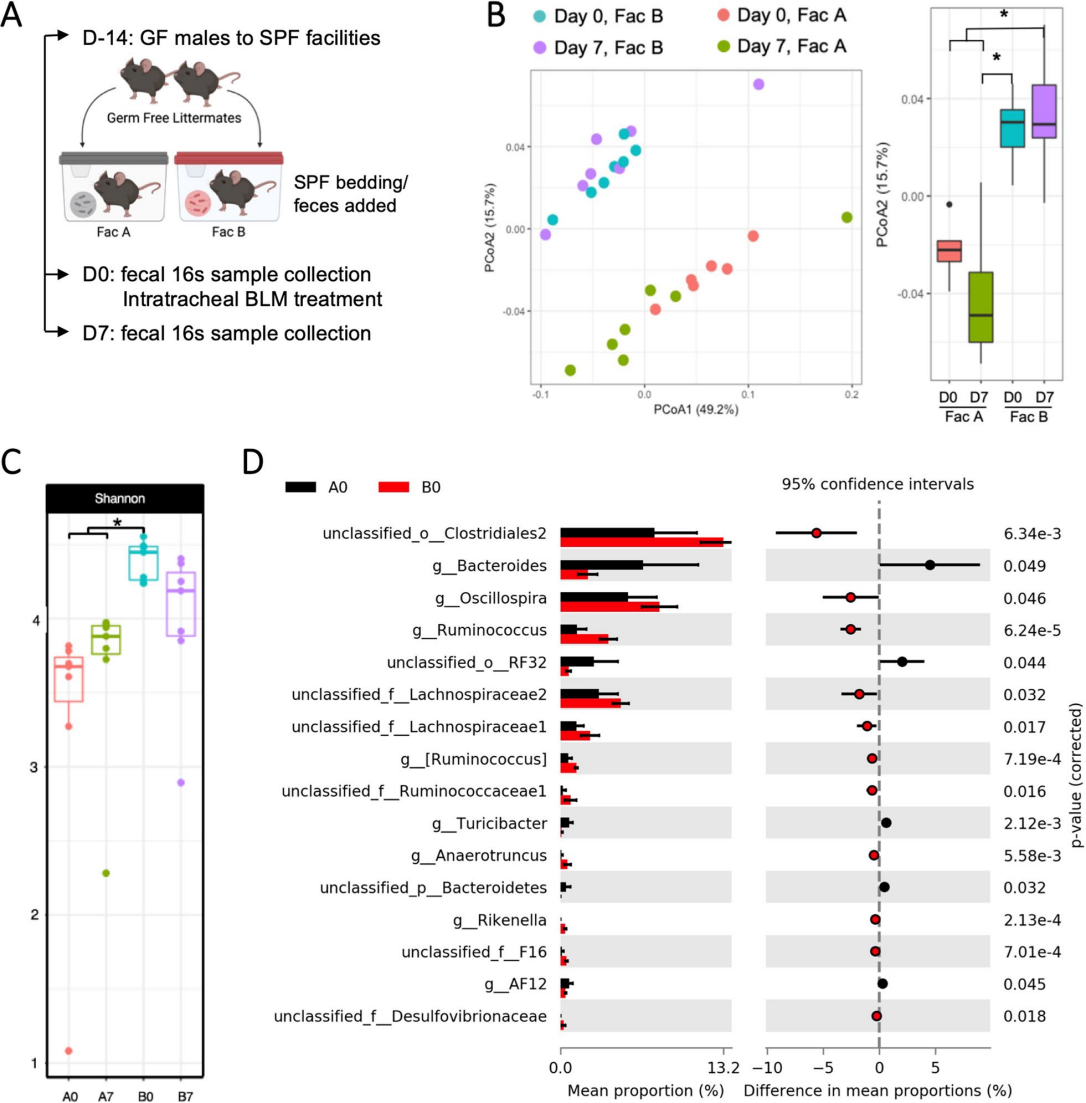
Gut microbial community structures are different between the two SPF facilities. **A** Following the 14-day conventionalization, fecal samples were collected from ex-GF mice at before (D0) and after (D7) bleomycin challenge (n = 7 per condition). Gut microbial structures were analyzed by 16S rRNA gene (V4 region) sequencing of the fecal samples. **B** Weighted UniFrac analysis of similarity coefficients were calculated from 16S rRNA gene sequences of each mouse. Permutational multivariate analysis of variance was done to analyze paired statistical differences in beta diversity. **C** The within-sample richness and evenness (alpha diversity) were measured by Shannon diversity index [A0 vs. B0 (p-value = 0.0003); A7 vs. B0 (p-value = 0.0267)]. **D** Differential abundance of microbial taxa between the two facilities at D0 was analyzed using the Statistical Analysis of Metagenomic Profiles (STAMP) package. P-value of 0.05 was used as a cutoff

To investigate whether the difference in BLM susceptibility among conventionalized ex-GF mice in the two SPF facilities can be attributed to microbial communities, we performed 16S rRNA gene amplicon sequencing of fecal samples obtained either at the time of (A0 and B0) or 7 days after (A7 and B7) the BLM challenge (Fig. 3A). Weighted UniFrac analysis of the amplicon sequence variants (ASV) showed that there was a significant difference in community structures of the fecal microbiomes in animals in the two facilities (Fig. 3B). The difference in weighted UniFrac distance between the two facilities was evident on the second principal component (PCoA2), independent of BLM challenge. Shannon index, which is a measure of total ASV number and the abundance of each ASV, was significantly higher in the samples from Facility B mice at baseline compared to that from Facility A mice at both baseline and 7 days after BLM challenge (Fig. 3C). These data suggest that not only are the overall structures of microbial communities different, but also the species diversity is greater in the microbial community in Facility B mice compared to Facility A mice.

To investigate whether specific taxa drive the difference in gut microbial diversity and structure, we compared the relative abundance of ASV by performing statistical analysis of metagenomic profiles (STAMP) on data from unperturbed animals. We found that members of *Ruminococcaceae* (family), *Lachnospiraceae* (family), *Oscillospira* (genus), *Rikenella* (genus), and *Desulfovibrionaceae* (family) were significantly more abundant in Facility B, whereas *Bacteroides* (genus) and *Turicibacter* (genus) were significantly enriched in Facility A (Fig. 3D). Three taxa with relatively high abundance in Facility B mice at baseline, including *Ruminococcaceae*, *Lachnospiraceae* and *Oscillospira*, are all members of the order *Clostridiales*. Additionally, some unclassified members of *Clostridiales2* were among the most differentially enriched taxa in Facility B mice.

Interestingly, we observed a shift in the abundance of *Clostridiales* after BLM treatment. Analyses of samples collected on day 7 after the BLM challenge revealed that the abundance of *Clostridiales2* was no longer significantly different, whereas some unclassified members of *Clostridiales1* (order) became significantly less abundant in Facility B mice compared to Facility A mice (Additional file 1: Fig. S2). Furthermore, there was enrichment of *Bifidobacterium* (genus), *Adlercreutzia* (genus), and *Turicibacter* (genus) in Facility A mice after BLM challenge. Interestingly, the significant enrichment of some members of *bacterium F16* (family) and *Desulfovibrionaceae* (family) among mice in Facility B was maintained even after BLM treatment (Additional file 1: Fig. S2). Overall, our analyses of the 16S rRNA gene sequence showed that the community structures and relative abundance of specific taxa are significantly different between genetically similar littermate mice that are conventionalized in two different facilities.

### Transfer of fecal microbiota between SPF mice in one housing facility to another modulates susceptibility to intratracheal BLM

**Fig. 4:**
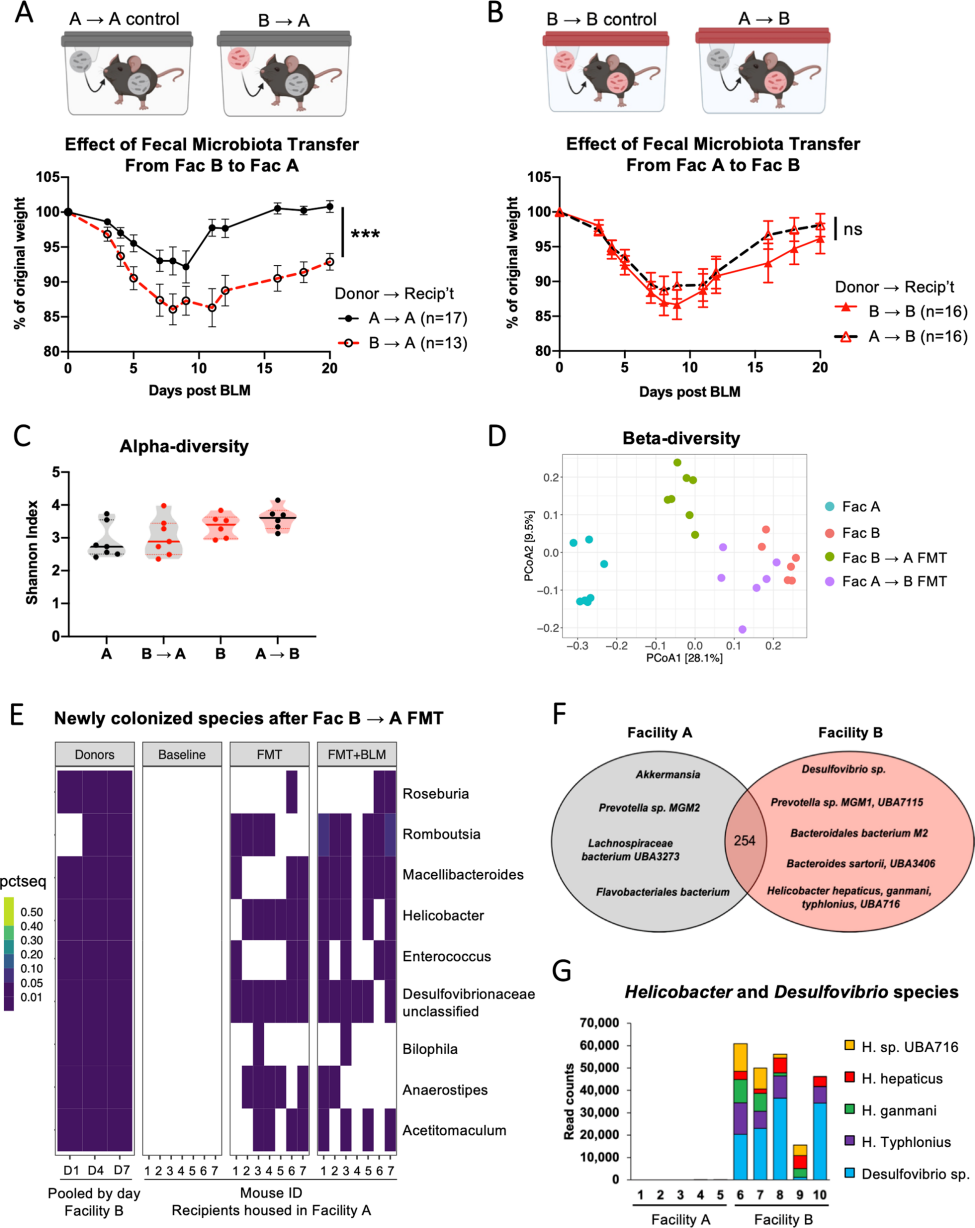
Additive fecal microbiota transfer from Facility B to A renders the recipients more susceptible to the lung injury response. **A**, **B** Littermate progenies of the conventionalized ex-GF founders that were born and raised in each SPF facility were separated into two cages. One cage of mice received the pooled fecal slurry from themselves (A → A or B → B), and another cage of mice received the pooled fecal slurry from mice housed in a different facility (A → B or B → A), via 3 oral gavages in a week. 10-days after the first dose of gavage, the mice were challenged with 1 U/kg of intratracheal bleomycin. Weight curves showing the effect of fecal microbiota transfer from the Facility B to A (**A**), and from the Facility A to B (**B**). Overall, around 70% of the mice from all groups survived with no statistically significant difference, and datapoints were censored upon death. P-values were obtained from the mixed-effects analysis of the data and depicted as P < 0.001 (***) or non-significant (ns). **C**–**E** Fecal samples collected from FMT recipients at before and after gavaging were sequenced for 16S rRNA genes (V4–V5 region). **C** The within-sample richness and evenness (alpha diversity) were measured by Shannon index. Four groups are A: samples collected from Facility A mice prior to FMT (n = 7), B → A: samples collected from Facility A mice at 10 days after the first dose of Facility B microbiota transplant (n = 7), B: samples collected from Facility B mice prior to FMT (n = 6), A → B: samples collected from Facility B mice at 10 days after the first dose of Facility A microbiota transplant (n = 6). **D** Unweighted UniFrac analysis of similarity coefficients were calculated from 16S rRNA gene sequences of each mouse. **E** Heatmap of the percent sequence amplicons measured from a list of commensal taxa that were present in donor mice in Facility B but absent in recipient mice in Facility A at baseline. Fecal samples from donor mice were pooled and sampled on days of gavage treatment. Fecal samples from 7 recipient mice were collected longitudinally at baseline, 10-days after the first dose of gavage (FMT), and 7-days after bleomycin challenge (FMT + BLM). **F**, **G** Shallow shotgun metagenomics analysis of fecal samples from progenies of the conventionalized ex-GF founder mice in the two facilities. The samples were collected from naïve mice without any treatment. Total 268 unique taxa were identified using k-mer based classification on One Codex databse (Transnetyx). **F** Venn diagram showing the taxa that were exclusively present in each facility. **G** Read counts for species within *Helicobacter* and *Desulfovibrio* genera in 10 mice sampled for metagenomic sequencing analysis

Given that unique microbiomes are maintained in each housing facility, we sought to determine whether each facility’s microbiome is associated with the promotion or prevention of lung injury. Although the differential BLM susceptibility phenomenon was initially observed throughout multiple inbred mouse strains in our animal facilities, some heterogeneity in the microbial community even in the same facility is inevitable due to the vertical transmission of mammalian gut microbiota from different ancestral origins [30]. To control for a possible impact of the ancestral microbiome, the progeny of conventionalized ex-GF littermates in each animal facility were maintained as stable SPF colonies for multiple generations and used for all following experiments in this study. To investigate whether the transfer of fecal microbiota from mice in one facility into mice housed in another facility can modulate susceptibility to BLM-induced lung injury, we performed fecal microbiota transfer (FMT) in these SPF mice. Without prior manipulation of the endogenous microbiome, SPF littermates in both facilities received FMT from animals in either the same facility or another facility via oral gavage 3 times, and then the mice were challenged with BLM. Strikingly, introducing the Facility B microbiota was sufficient to increase the weight loss of SPF animals in Facility A, compared to their littermate controls, in response to the BLM challenge (Fig. 4A). However, the Facility A microbiota did not change the BLM-induced weight loss of animals in Facility B (Fig. 4B). These data suggest that some components of Facility B microbiota are responsible for worsening the BLM-induced weight loss in mice in a dominant-negative manner.

### Colonization of *Helicobacter* and *Desulfovibrio* species are associated with increased weight loss during ALI response

To ensure the colonization of transplanted microbiome, we characterized fecal microbiomes of the mice at both baseline and after FMT by 16S rRNA gene amplicon sequencing. Alpha-diversities of the microbiome in FMT recipient animals were comparable to those of control animals, despite the introduction of additional microbial species (Fig. 4C). Unweighted UniFrac analysis revealed that the microbial community structures of mice from the two facilities were distinct at baseline, but they became more similar to the respective donor microbiomes after FMT (Fig. 4D). Facility B microbiota recipients (Fac B → A FMT), which showed the most significant change in lung injury response, had the greatest shift in PCoA1 of their fecal microbiomes. Since UniFrac takes the phylogenetic distance into account [21], the drastic difference in the beta diversity could be driven by the engraftment of some phylogenetically distant lineages by FMT.

Using 16S rRNA gene analysis, we investigated whether the Facility B donor-specific taxa can successfully colonize the FMT recipients in Facility A. Based on the percent sequence reads, we found that two members of proteobacteria, *Helicobacter* and *Desulfovibrionaceae*, were among the most consistently colonized genera (Fig. 4E and Additional file 1: Fig. S3) across biological replicates. Importantly, several donor-specific taxa also showed successful engraftment in the Facility A microbiota recipients (Fac A → B FMT) despite driving a small shift in PCoA1. The newly introduced microbes from Facility A were assigned to multiple phyla, including *Vampirovibrio*, *Ruminococcus*, *Alloprevotella*, *Allobaculum*, and *Akkermansia* (Additional file 1: Fig. S4)*.* These findings suggest that the Facility A microbiota is unable to suppress the negative effect of pre-existing microbiota in Facility B mice, but Facility B microbiota can dominantly worsen the BLM-induced weight loss in Facility A mice.

To further elucidate the microbes that may be responsible for promoting severe weight loss in Facility B mice, we performed shotgun metagenomic sequencing of fecal microbiota from naïve mice in the two facilities. A total of 268 taxa were identified from fecal sample sequences. The normalized reads for virus, fungi, archaea, and protists were less than 0.005%, and there was no observable difference in compositions of these non-bacterial taxa between animals from the two facilities. Similar to the results of differentially colonized taxa in FMT recipients, we found members of both *Desulfovibrio* and *Helicobacter* genera to be exclusively present in Facility B microbiota at baseline (Fig. 4F). With the greater resolution provided by metagenomic sequencing, we were able to identify candidate microbes at the species level. The microbial species associated with severe weight loss in Facility B included *Desulfovibrio* sp. (Tax ID 885), *Helicobacter hepaticus* (Tax ID 32025), *H. ganmani* (Tax ID 60246), *H. typhlonius* (Tax ID 76936), and *H. UBA716* (Tax ID 1946589), all of which were absent in the Facility A microbiota (Fig. 4G). Thus, our results from both 16S rRNA gene and metagenomic sequencing suggest that *Desulfovibrio* and *Helicobacter* species are candidate microbes that modulate the degree of weight loss during BLM-induced lung injury.

### Naïve SPF mice housed in Facility A and Facility B have unique immunophenotypes

**Fig. 5:**
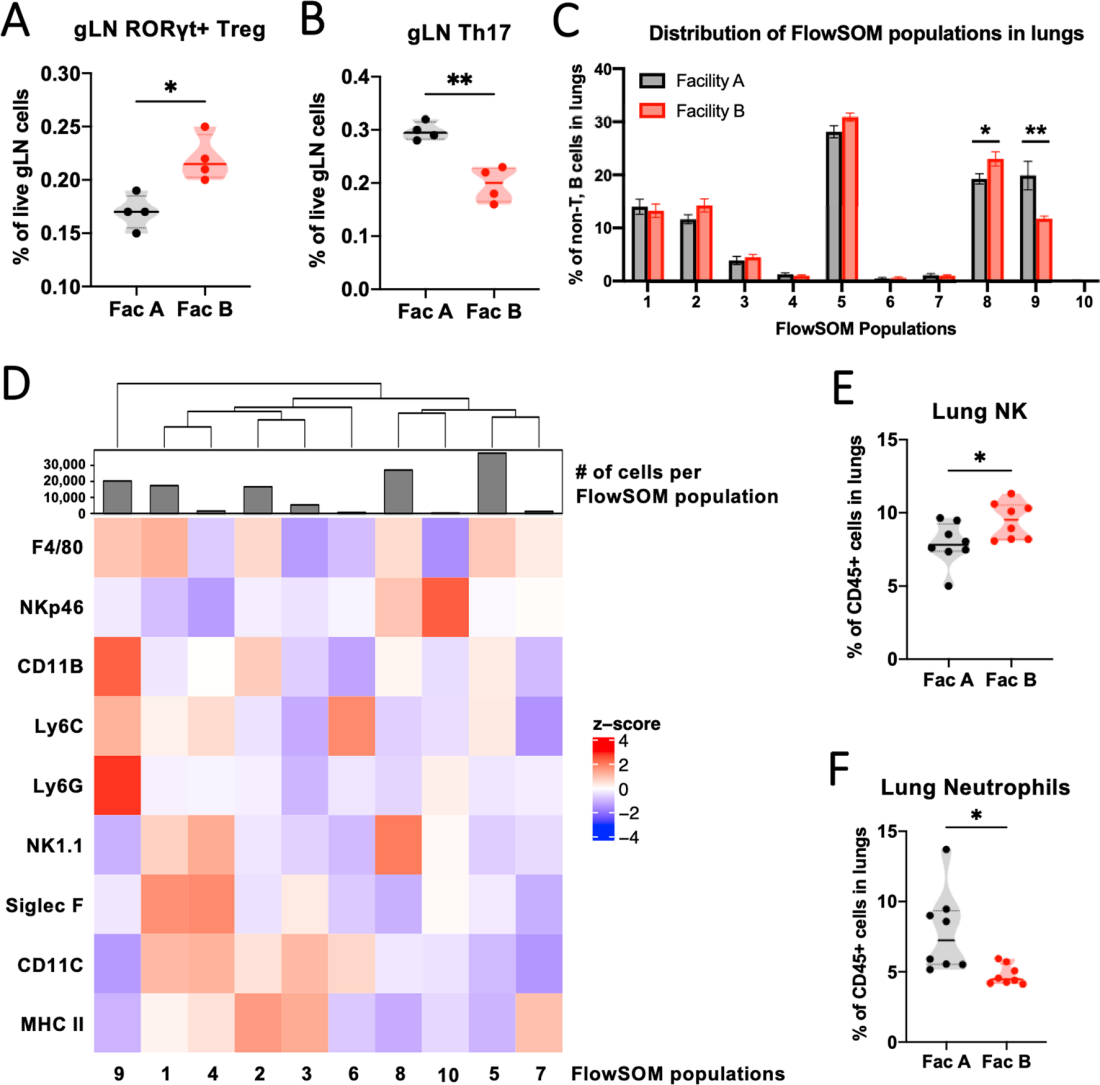
Differential colonization of commensal species corresponds with specific immune landscapes of the lungs in naïve animals. Gut-draining LN and lungs of unperturbed progenies of the conventionalized ex-GF founders were immunophenotyped. **A** Frequencies of RORγt+ Treg (CD25+Foxp3+) cells in gLN. **B** Frequencies of Th17 (RORγt+Foxp3−) cells in gLN. **C**, **D** Non-adaptive immune cell population (CD45+CD3−CD19−) in the lungs were analyzed using the FlowSOM clustering algorithm. Total 128,000 cells were analyzed from n = 8 mice from each facility (Down sampled to 8000 cells per mouse). FlowSOM generated 10 clusters with unique phenotypes. **C** Proportion of cells in each cluster is compared between mice in the two facilities. Multiple unpaired t-tests using the two-stage step-up method were performed for statistical analysis. **D** Heatmap of expressions of 9 markers in each cluster. **E** Frequencies of NK cells in the lungs based on manual gating (NK1.1+lineage−). **F** Frequencies of neutrophils in the lungs (Ly6G+CD11b+lineage−). P-values were obtained from student t-tests and depicted as P < 0.05 (*), P < 0.01 (**), P < 0.001 (***)

The gut microbiota plays an essential role in the development and maintenance of the host’s immune system, which can determine the health of not only the gut, but also distant organs including the lungs. To test the hypothesis that SPF mice housed in the two facilities have distinct baseline immunophenotypes, we designed comprehensive flow cytometry panels for T cells, dendritic cells, B cells, innate immune cells, and innate-like lymphocytes and analyzed gut-draining lymph nodes (gLN), spleens, and lungs of age- and sex-matched naïve animals from the two facilities. First, we focused on investigating phenotypes of T cells in the gLN as some commensal microbes in our housing facilities are known to regulate T helper cell differentiation. As *H. hepaticus* is a pathobiont, WT mice have adapted a tolerance mechanism, which is induction of RORγt+Foxp3+ regulatory T (Treg) cells that suppress pro-inflammatory T helper 17 (Th17) cells [31, 32]. Consistent with this, we found that RORγt+ Treg cells were increased and Th17 cells were decreased in gLN of the *H. hepaticus*-harboring mice in Facility B compared to the Facility A mice at baseline (Fig. 5A, B, Additional file 1: Fig. S5A). However, compositions of adaptive immune cells in the lungs were comparable between naïve mice in the two housing facilities (Additional file 1: Table S1).

Next, we performed FlowSOM clustering analysis to compare overall landscapes of the innate immune cells in the lungs of naïve mice in the two facilities. We found that FlowSOM population 8 was increased, and population 9 was decreased in Facility B mice compared to Facility A mice (Fig. 5C). Cells in population 8 expressed markers consistent with NK cells, and neutrophil markers were expressed in population 9 (Fig. 5D). Based on the manual gating, we confirmed that the proportion of NK cells was increased, and neutrophils were decreased in the lungs of Facility B mice (Fig. 5E, F, Additional file 1: Fig. S5B, C). Interestingly, the frequency of neutrophils was also lower in the spleens of Facility B mice, further corroborating our results (Additional file 1: Fig. S5D). This suggests that granulopoiesis during homeostasis might be differentially regulated in these SPF mice with distinct gut microbiota. Overall, our data demonstrate that mice housed in the two facilities have distinct immunophenotypes, such that the gut lymph node Th17 cells and systemic neutrophils are downregulated in Facility B mice at baseline.

### Neutrophil recruitment to the lungs is more robust in Facility B mice during BLM-induced lung injury response

**Fig. 6:**
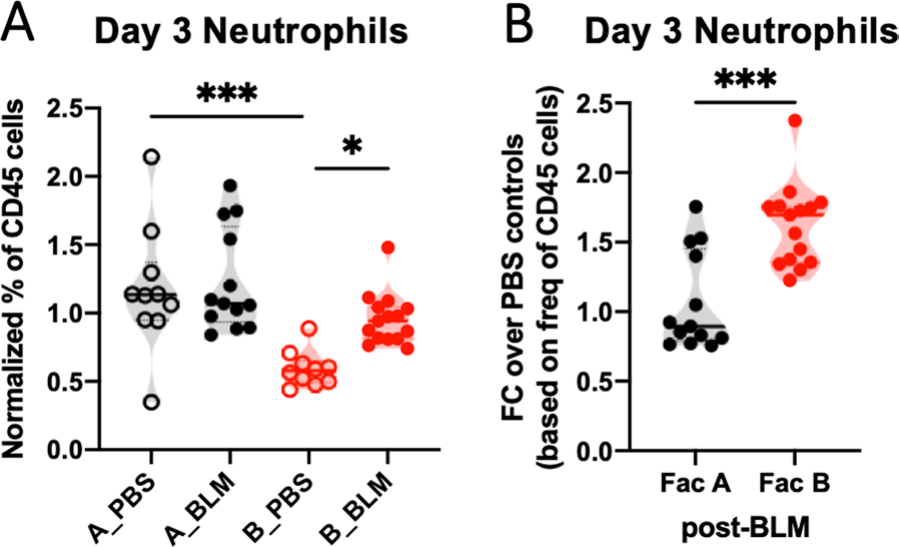
Relatively increased neutrophil recruitment to the lungs during lung injury coincides with worse weight loss in mice in Facility B. Lungs were harvested from progenies of the conventionalized ex-GF founders in the two facilities after bleomycin (1 U/kg) or PBS (vehicle control) challenge. **A** Normalized frequencies of neutrophils in the lungs on day 3 post-BLM. Data from three independent experiments were combined, and experimental values were normalized by the group variance for each experiment. The cells were gated based on Ly6G+CD11b+lineage− as shown in Additional file 1: Fig. S5C. P-values were obtained from Sidak’s multiple comparisons test after confirming P < 0.05 from one-way ANOVA. **B** Fold change of neutrophil accumulations in lungs on 3 days after bleomycin challenge compared to PBS treatment in each facility. P-values are depicted as P < 0.05 (*), P < 0.01 (**), P < 0.001 (***)

To investigate whether the cellular responses during BLM are different between animals housed in the two facilities, we immunophenotyped lungs of mice at 3 days post-BLM administration. Facility B mice were more sensitive to BLM treatment and recruited more neutrophils to the lungs during the acute phase of injury, even though control PBS treated mice in Facility B maintained neutrophils at lower levels than Facility A control mice (Fig. 6A). On day 3 after BLM treatment, lung neutrophils were increased by over 1.5-fold after BLM treatment in Facility B mice, compared to little change in Facility A mice (Fig. 6B). By day 6 post-BLM, neutrophils were recruited at similar levels in lungs of mice in both housing facilities (data not shown). Histopathological analysis of lung sections showed that BLM-treated lungs of mice in both facilities have similar patterns of patchy neutrophilic alveolitis as previously described [8] (Additional file 1: Fig. S6). Therefore, increased neutrophil numbers at homeostasis may confer protection from BLM-induced weight loss, while a rapid fold increase in neutrophils during BLM response is detrimental.

## Discussion

Our study demonstrates that the composition of commensal fecal microbiota impacts the severity of BLM-induced weight loss in C57BL/6 mice. Using various mouse strains that we maintain in two animal housing facilities on campus, we observed that BLM-challenged WT littermate control mice have strikingly high mortality in one facility (Facility B), compared to the mice in another facility (Facility A). We determined that WT mice maintain distinct gut microbial communities based on housing facilities in which the mice were born and raised, despite both facilities having the same SPF protocols. Further, unperturbed WT mice in Facility B had lower Th17 and neutrophil levels compared to Facility A mice. During the BLM challenge, Facility B mice responded with a greater fold increase in lung neutrophils, indicating a more severe inflammatory response. We demonstrate that the BLM-highly-susceptible phenotype is transferrable by the fecal microbiota. When the two microbial communities are combined, the Facility B-associated microbiota exerts a dominant-negative effect. We identify members of *Helicobacter* and *Desulfovibrio* genera as candidate microbes that contribute to the increased susceptibility to BLM-induced weight loss.

While the initial observation was on survival difference between various B6 strains housed in the two facilities, the effect of the microbiota on genetically identical animals and their progenies became largely evident by the degree of weight loss rather than survival. Weight loss and survival are directly linked because animal death in our survival study was mostly due to excessive weight loss. The less dramatic phenotypes in conventionalized GF mice and their SPF progenies are likely attributable to changes in microbiota compositions over the years of this study. Both facilities are maintained as level I barriers, which require constant monitoring for pathogens, personal protective equipment, and biosafety cabinet usage. Since there is no hierarchical entry restriction for these facilities, the relative abundance of some taxa could have been shifted over time. Therefore, we defined candidate microbes for body weight modulation during BLM-challenge by identifying the consistent differences between the two facilities in three independent sets of fecal microbiome sequencing data, which were collected at three different time periods. Notably, *Desulfovibrio* and *Helicobacter* always remained excluded from Facility A, and their presence in Facility B consistently correlated with aggravated weight loss following BLM during the past several years of the study period.

Our data suggest potential pro-inflammatory roles for *Desulfovibrio* sp. and some *Helicobacter* species during the lung injury response. *Desulfovibrio* sp. is an unknown species of the *Desulfovibrio* genus, which is a group of sulfate-reducing bacteria found in the gastrointestinal tracts of many animals including humans [33, 34]. Little is known about this commensal species, but other related species of *Desulfovibrio* have recently been discovered to be associated with rare cases of bacteremia [35–37]. On the other hand, the immune impact of *H. hepaticus* has been well-documented in the context of gut health and disease. *H. hepaticus* is a pathobiont, which can cause colitis in *Il10*-deficient mice only in specific microbial environments [32, 38–40]. Interestingly, gut colonization with *H. hepaticus* has been shown to promote persistent lung injury after *Mycobacterium tuberculosis* infection in mice [41], suggesting that crosstalk between *H. hepaticus* and the host immune system can modulate immune responses in the lungs. In support of this idea, we found that the proportion of RORγt+ Treg cells is increased in gLN of naïve mice in Facility B, where *Helicobacter* species are present. Furthermore, the relative proportion of neutrophils is decreased in both lungs and spleens of BLM-highly-susceptible animals at baseline. IL-17A signaling via G-CSF is a key mediator of granulopoiesis and neutrophil recruitment [42, 43], and RORγt+ Treg cells can specifically suppress Th17 cells, which secrete IL-17A [31]. Therefore, the systemic decrease in neutrophils that we observed in BLM-highly-susceptible animals at homeostasis could be explained by the expansion of Th17-specific immunoregulatory cells.

The implication of a decreased Th17-neutrophil axis at homeostasis in BLM-highly-susceptible mice is puzzling. Activated neutrophils during ALI play pathogenic roles by releasing proinflammatory cytokines and cytotoxic products, including granular enzymes, reactive oxygen species, and neutrophil extracellular traps [44–47]. Our finding that the Facility A mice showed no increase in lung neutrophils, while Facility B mice had over 1.5-fold increase in lung neutrophils in only 3 days after the BLM challenge is consistent with the known role of neutrophils in lung injury. However, the Facility B mice had relatively smaller proportions of neutrophils in the lungs at both baseline and during intratracheal PBS vehicle treatment. We postulate that increased neutrophils at homeostasis might be protective, and the gut microbiota may modulate neutrophils at homeostasis. It has been shown that the level of granulopoiesis during steady state is influenced by the microbiota [48, 49]. Further, some gut commensals can promote change in lung neutrophil phenotypes to provide protection from lung infection [50, 51]. Thus, while mechanisms involved in the potentially protective role of neutrophils remain unclear, it’s possible that the gut microbiota modulate granulopoiesis and neutrophil functions.

The effect of fecal microbiota transplant on lungs could be both systemic and lung specific. Since we transferred entire fecal contents, the effect may be systemic partially due to some metabolites and other secretory factors present in the slurry. For example, bacterial metabolites called short-chain fatty acids (SCFAs) are known to maintain epithelial barrier integrity and protect against allergic inflammation in the lung [52]. Further, the level of fecal secretory IgA (sIgA), which can be regulated by members of *Sutterella* genus microbes in the gut, determines the severity of dextran sodium sulphate-induced intestinal injury [53]. These secretory factors in circulation could impact health of structural cells throughout the body, including the lungs. On the other hand, perturbation of the gut microbiota could affect the lungs via the gut-lung axis. Correlations observed between gut microbiome composition and respiratory disease development support the idea of crosstalk between the two organs [54, 55]. Microbial communities in the gut and lungs have shown to be interconnected from the time of embryonic developmental in both health and disease [56, 57]. Furthermore, some gut microbes are abundantly found in the lungs and correlated with severity or development of ALI/ARDS in experimental models of sepsis and studies of mechanically-ventilated patients, respectively [4, 7]. Finally, while we delivered the fecal microbiota directly into the gut by oral gavage, we cannot eliminate the possibility that we also introduced gut microbes to the lungs through translocation and aspiration of the donor microbiota.

One factor that may explain the difference in the overall microbial community structures between the two housing facilities is that the mice were on different standard chow diets. This difference in diets were already in place prior to the present study, and hence, could be contributing to our findings. BLM-highly-susceptible mice in Facility B were on Teklad Global 18% Protein Rodent Diet (2918, Harlan-Envigo) throughout their lifetime including during fetal development. However, breeders in Facility A were on a high-fat diet, and all other mice were given NIH-31 Modified Open Formula (7913, Harlan-Envigo) after weaning. Both standard non-high fat diets 2918 and 7913 consist of similar macronutrient compositions and are irradiated. Differences between the two diets are that the 7913 diet (Facility A) contains ground oats, fish meal, and dehydrated alfalfa meal, whereas the 2918 diet (Facility B) contains l-lysine and dl-methionine. While diets would not be the source of new commensal species, the subtle differences in ingredients could select for specific taxa and contribute to maintaining the unique microbial communities between the two facilities.

Our results support the role for microbiota in controlling the BLM-induced injury response. Of note, the impact of gut microbiota on response to BLM challenge seems to be most significant during the acute phase of BLM treatment, while a previous work has shown that the gut-specific depletion of microbiome has a negligible impact on development of BLM-induced pulmonary fibrosis [14]. We demonstrate the need for controlling the presence of *Proteobacteria* in mice microbiota during the study of lung injury response. Notably, *H. hepaticus* and *Desulfovibrio* are not among the excluded pathobionts in specific pathogen-free facilities and vendors unless specifically requested. Our study highlights a need for reporting the composition of commensal microbiomes in laboratory animals, in order to improve reproducibility in research using the BLM model across institutions. Our data also impacts the potential use of BLM in cancer patients as it is a chemotherapy that is effective but less-often used due to infrequent, but serious side effects including lung pneumonitis and fibrosis. As the development of lung complications is also unpredictable in cancer patients, understanding whether the microbiome of each patient affects their chances of developing pneumonitis may influence clinical decision-making.

## Supplementary Information

**Additional file 1: Fig S1.** Intratracheal LPS-induced ALI responses in mice housed in the two facilities. **Fig S2.** 16S rRNA sequencing of conventionalized ex-GF mice on D7 after BLM challenge. **Fig S3.** Full list of taxa resolved from fecal samples from donor and recipient mice in the Facility B-to-A FMT experiment. **Fig S4.** Unchanged lung injury outcome in Fac A microbiome recipients is not due to the lack of colonization of additional species. **Fig S5.** Examples of flow cytometry gating strategies and frequencies of spleen neutrophils in naive animals. **Fig S6.** Hematoxylin and eosin (H&E) staining of a mouse lung section on day 3 post-BLM challenge. **Table S1.** Frequencies of innate and adaptive immune cell populations in the lungs from unperturbed 15–17 weeks old animals raised in the respective housing facilities. **Table S2.** List of antibodies used for flow cytometry analysis.

## Data Availability

All 16s rRNA amplicon and metagenomics sequencing data files are available in the NCBI Sequence Read Archive (Accession: PRJNA903920).

## Abbreviations

BLM: Bleomycin
ALI: Acute lung injury
ARDS: Acute respiratory distress syndrome
SPF: Specific pathogen-free
GF: Germ-free
WT: Wild type
FMT: Fecal microbiota transfer
ASV: Amplicon sequence variants
PCoA: Principal coordinate analysis
STAMP: Statistical analysis of metagenomic profiles
gLN: Gut-draining lymph nodes
Treg: Regulatory T cells
Th17: T helper 17 cells
