# Supplementary material for: Gut microbiota modulates bleomycin-induced acute lung injury response in mice
Source: Respir Res. 2022 Dec 10;23:337. doi: 10.1186/s12931-022-02264-7 (PMC9741526; doi:10.1186/s12931-022-02264-7)
Supplement: Supplementary file 1 — Additional file 1: Fig S1. Intratracheal LPS-induced ALI responses in mice housed in the two facilities. Fig S2. 16S rRNA sequencing of conventionalized ex-GF mice on D7 after BLM challenge. Fig S3. Full list of taxa resolved from fecal samples from donor and recipient mice in the Facility B-to-A FMT experiment. Fig S4. Unchanged lung injury outcome in Fac A microbiome recipients is not due to the lack of colonization of additional species. Fig S5. Examples of flow cytometry gating strategies and frequencies of spleen neutrophils in naive animals. Fig S6. Hematoxylin and eosin (H&E) staining of a mouse lung section on day 3 post-BLM challenge. Table S1. Frequencies of innate and adaptive immune cell populations in the lungs from unperturbed 15–17 weeks old animals raised in the respective housing facilities. Table S2. List of antibodies used for flow cytometry analysis. [file 12931_2022_2264_MOESM1_ESM.pdf]

Gut microbiota modulates bleomycin-induced acute lung injury response in mice

Young me Yoon, Cara L Hrusch, Na Fei, Gabriel M Barrón, Kathleen AM Mills, Maile K Hollinger, Tania E Velez, Vanessa A Leone, Eugene B Chang, Anne I Sperling

Additional file 1

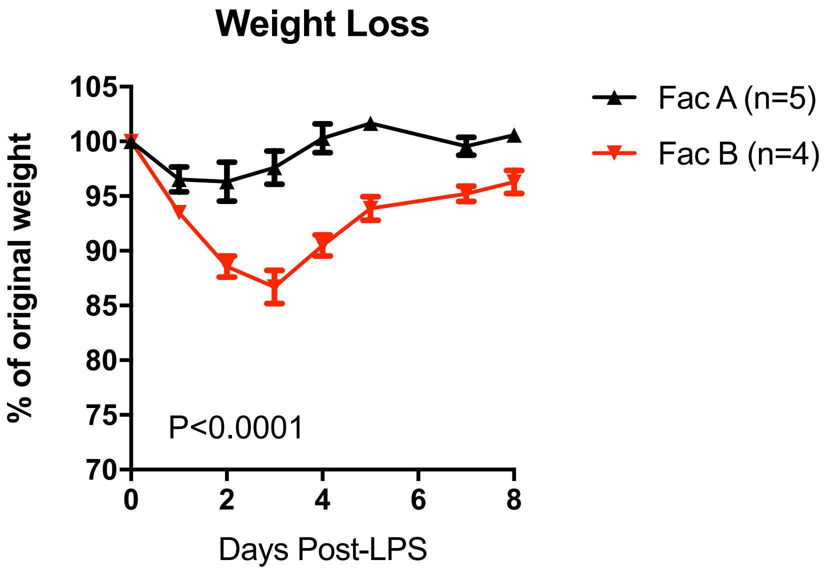

Fig S1. Intratracheal LPS-induced ALI responses in mice housed in the two facilities. Mice were challenged with 1mg/kg LPS. 2-way ANOVA was performed to assess statistical significance.

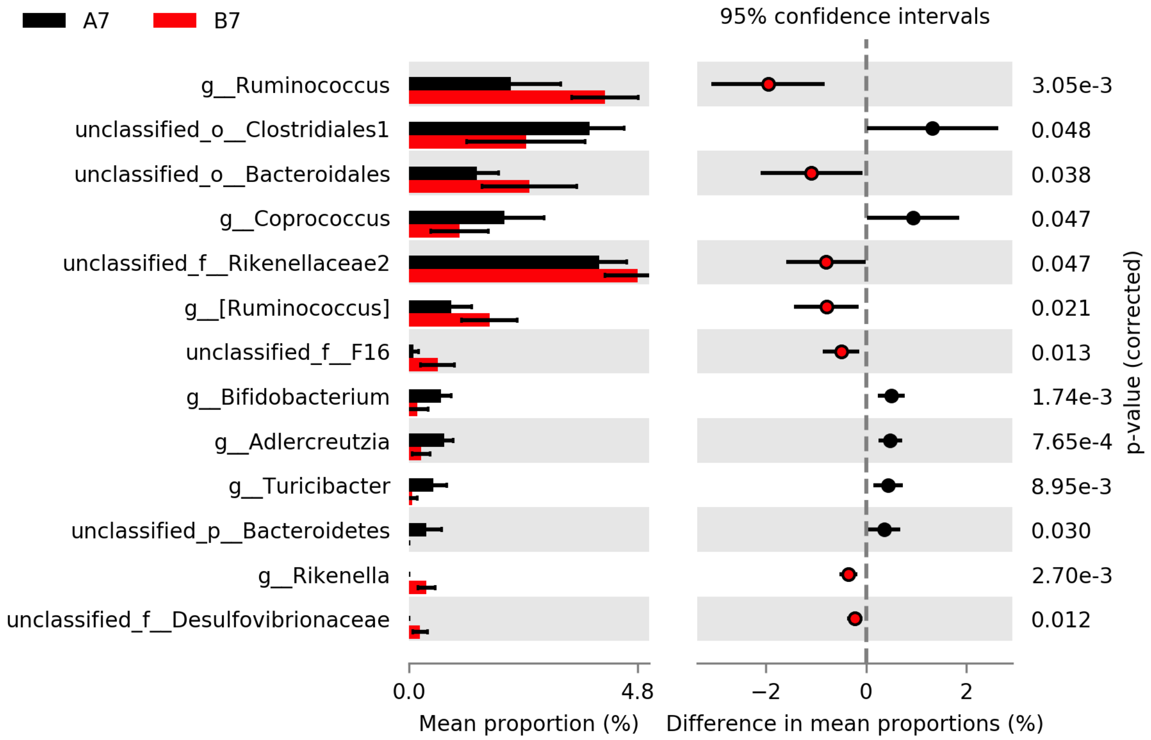

Fig S2. 16S rRNA sequencing of conventionalized ex-GF mice on D7 after BLM challenge.
Differential abundance of fecal microbial taxa between animals in the two facilities at 7-days post BLM treatment was analyzed using the Statistical Analysis of Metagenomic Profiles (STAMP). P-value of 0.05 was used as a cutoff.

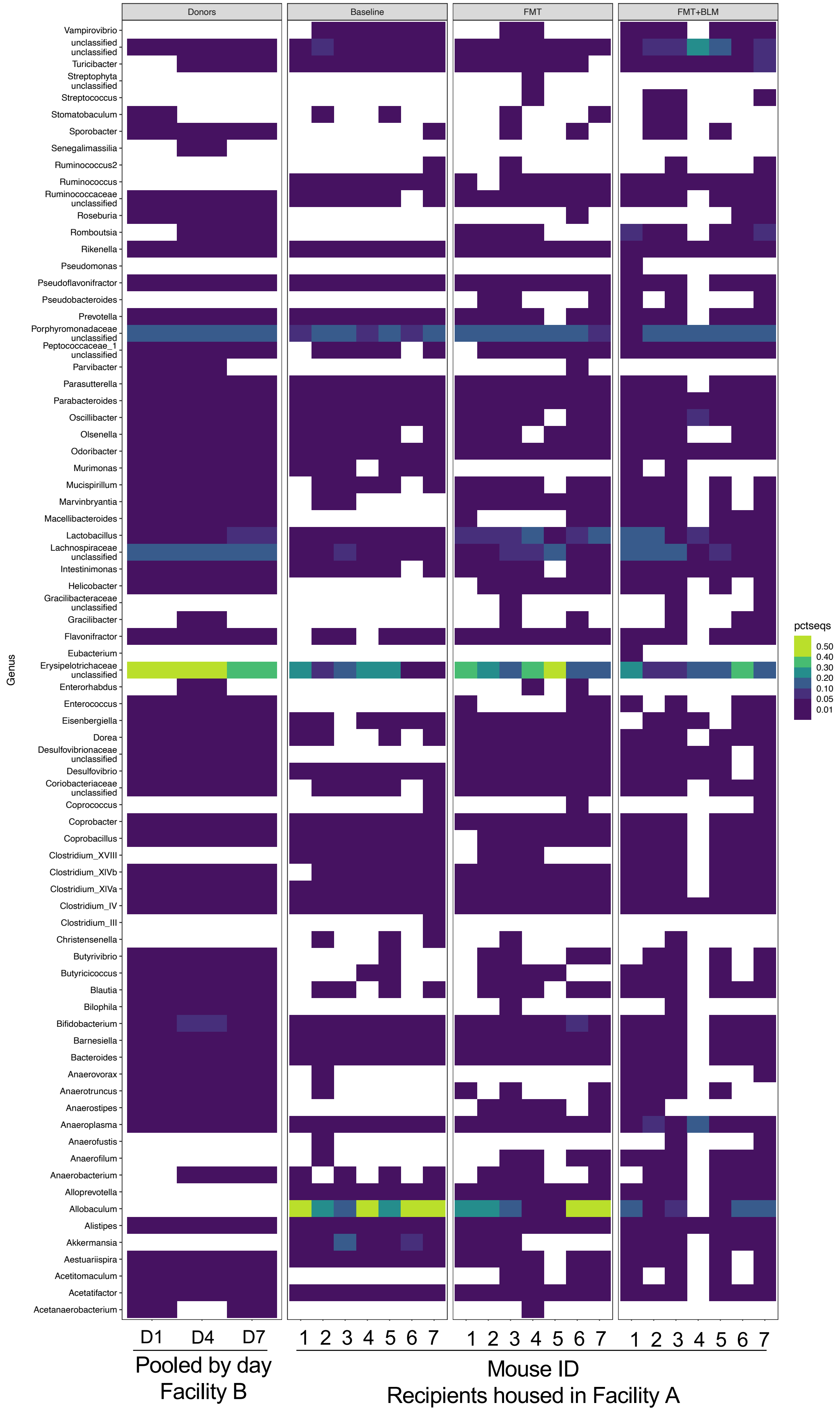

Fig S3. Full list of taxa resolved from fecal samples from donor and recipient mice in the Facility B-to-A FMT experiment. Heatmap visualizing percent sequences of all the taxa resolved to genus level. Fecal samples from donor mice of Facility B microbiota were pooled and sampled on each day of gavage treatment. Fecal samples from 7 recipient mice in Facility A were collected longitudinally at baseline, 10-days after the first dose of gavage (FMT), and 7-days after bleomycin challenge (FMT+BLM).

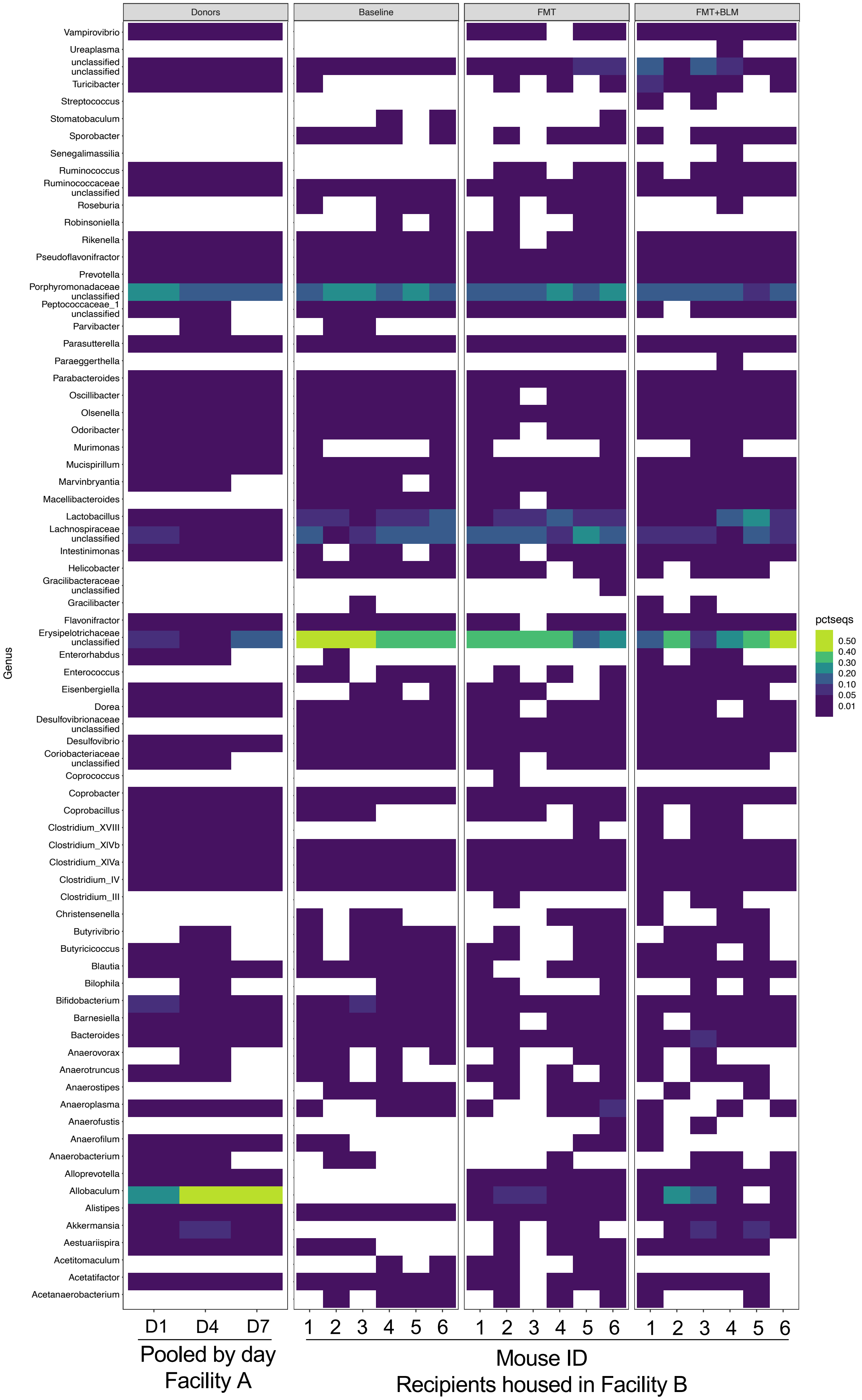

Fig S4. Unchanged lung injury outcome in Fac A microbiome recipients is not due to the lack of colonization of additional species. Heatmap visualizing percent sequences of all the taxa resolved to genus level. Fecal samples from donor mice of Facility A microbiota were pooled and sampled on each day of gavage treatment. Fecal samples from 6 recipient mice in Facility B were collected longitudinally at baseline, 10-days after the first dose of gavage (FMT), and 7-days after bleomycin challenge (FMT+BLM).

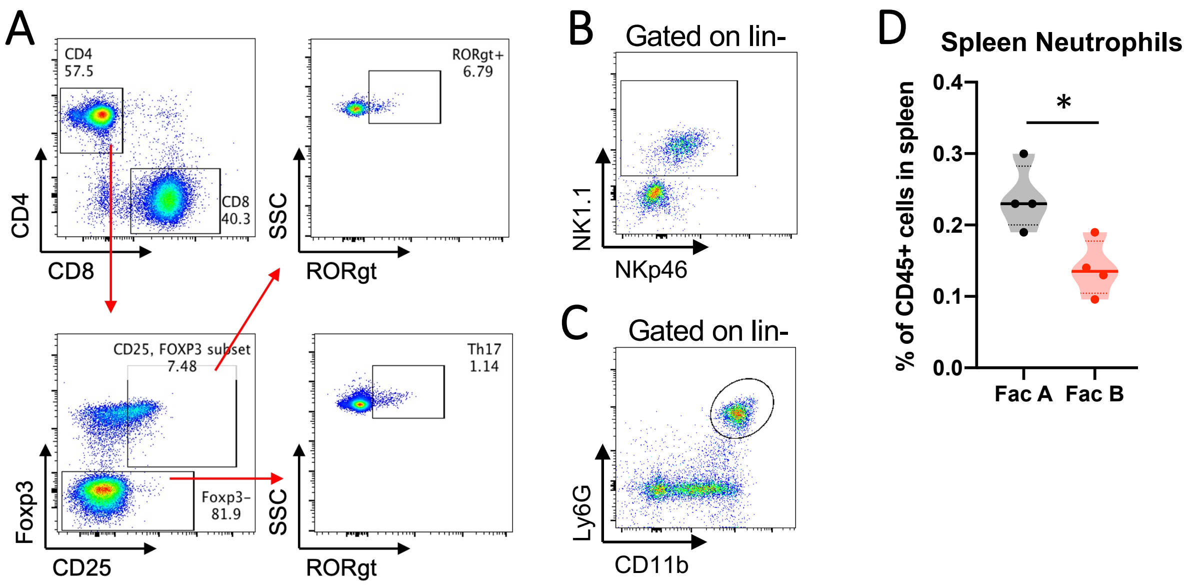

Fig S5. Examples of flow cytometry gating strategies and frequencies of spleen neutrophils in naïve animals. (A) Gating strategies for RORγt+ CD25+ Treg and Th17 populations, using gLN cells as representative. (B) Gating strategy for NK cells in lungs. Cells were pre-gated on CD45+, TCRb-, CD19-, non-AM, non-neutrophil, non-Eosinophil, non-DC population. (C) Gating strategy for neutrophils in lungs. Cells were pre-gated on CD45+, TCRb-, CD19-, non-AM population. (D) Frequency of neutrophils in spleens of unperturbed animals housed in the two facilities. T-test was done to obtain the p-value.

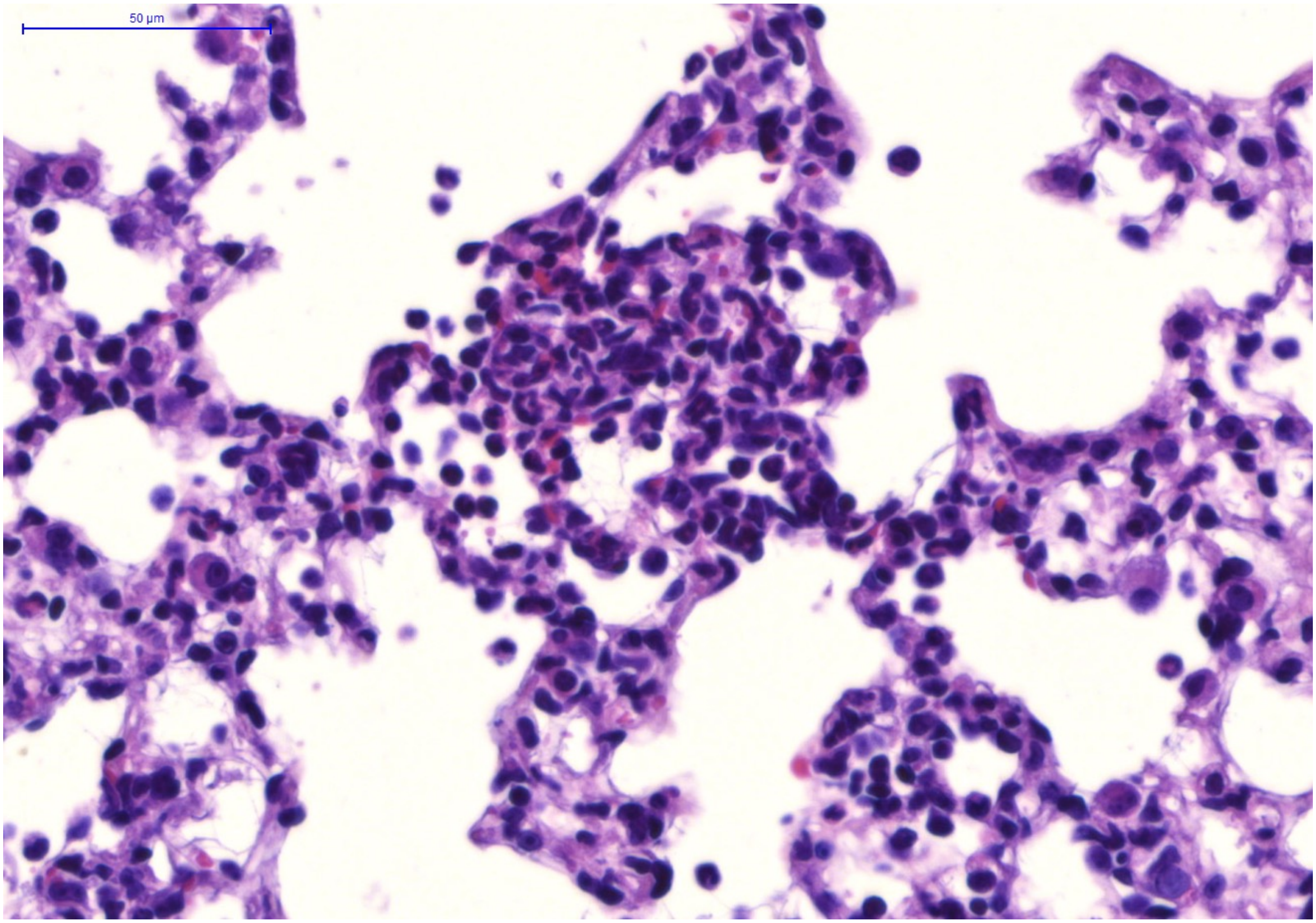

Fig S6. Hematoxylin and eosin (H&E) staining of a mouse lung section on day 3 post-BLM challenge. An example of patchy neutrophilic alveolitis that are found in BLM-treated mice in both housing facilities. The image shown here is from a Fac B mouse, but a similar histopathological pattern was observed in Fac A mice. For staining, left lungs harvested from mice on day 3 post-BLM treatment were formalin fixed and paraffine embedded. Lung blocks were sectioned at 5µm thick and stained with H&E. Slides were imaged with a 40x objective using the CRi Pannoramic SCAN. The scale bar is 50µm long.

Table S1. Frequencies of innate and adaptive immune cell populations in the lungs from unperturbed 15-17 weeks old animals raised in the respective housing facilities (n=8 per facility). Values are the percent of CD45+ cells (± standard deviation), and p-values are from t-test.

Lung immunophenotypes at baseline. Values as percent of CD45+ (± standard deviation)			
Cell population	Fac A	Fac B	P-value
Neutrophils	7.86 (±2.94)	4.78 (±0.71)	0.01
Eosinophils	2.87 (±0.66)	3.39 (±1.17)	0.29
DC	5.13 (±1.45)	6.46 (±1.47)	0.09
Monocyte	12.07 (±1.86)	13.76 (±0.97)	0.04
NK cells	7.88 (±1.46)	9.46 (±1.27)	0.04
B cells	36.45 (±7.57)	36.04 (±4.27)	0.90
$T_{\gamma\delta}$ cells	5.16 (±0.75)	5.33 (±0.54)	0.62
$T_{\alpha\beta}$ cells	19.58 (±4.06)	20.04 (±2.26)	0.78
CD8 T cells	4.14 (±0.49)	4.78 (±1.01)	0.13
CD4 T cells	10.06 (±2.64)	9.23 (±1.19)	0.43
T_{conv}	8.60 (±2.32)	7.85 (±1.17)	0.43
Th1	1.29 (±0.53)	1.40 (±0.60)	0.69
Th2	0.42 (±0.23)	0.42 (±0.17)	0.93
Th17	0.53 (±0.47)	0.23 (±0.07)	0.10
T_{reg} (all Foxp3+)	1.41 (±0.36)	1.31 (±0.22)	0.53
TBET+ T_{reg}	0.13 (±0.04)	0.15 (±0.09)	0.52
GATA3+ T_{reg}	0.17 (±0.09)	0.18 (±0.07)	0.79
RORγt+ T_{reg}	0.07 (±0.04)	0.07 (±0.03)	0.91
CD25+ T_{reg}	0.65 (±0.18)	0.60 (±0.11)	0.52
TBET+ CD25+ T_{reg}	0.04 (±0.02)	0.05 (±0.06)	0.49
GATA3+ CD25+ T_{reg}	0.08 (±0.05)	0.08 (±0.04)	0.83
RORγt+ CD25+ T_{reg}	0.03 (±0.02)	0.04 (±0.02)	0.29

Table S2. List of antibodies used for flow cytometry analysis

Antigen	Fluorophore	Clone	Company	Dilution Factor
CD3	BUV395	17A2	BD	200
CD4	BUV805	GK1.5	BD	200
CD44	Pacific Blue	IM7	BioLegend	400
CD8	BV510	53-6.7	BioLegend	200
CD45	BV605, BV785, BUV805	30-F11	BioLegend	200
CD62L	BV711	MWL-14	BioLegend	200
CD25	BV786	3C7	BD	100
TCRgd	APC-Fire750	GL3	BioLegend	200
Helios	FITC	22F6	BioLegend	400
RORgt	PerCP-Cy5.5	Q31-378	BD	100
EOMES	PE	Dan11mag	Invitrogen	400
T-bet	PE-Cy7	4B10	BioLegend	200
Foxp3	APC	FJK-16s	Invitrogen	400
GATA3	PE-CF594	L50-823	BD	50
NK1.1	FITC	PK136	BioLegend	400
MHC II	PerCP-Cy5.5	M5/114.15.2	BioLegend	400
Ter119	APC	TER-119	BioLegend	200
F4/80	APC-eFluor780	BM8	eBioscience	200
NKp46	BV421	29A1.4	BioLegend	50
CD11B	BV510, PE-dazzle	M1/70	BioLegend	400
Ly6C	BV605, APC-cy7	HK1.4	BioLegend	200
Ly6G	BV711	1A8	BioLegend	200
CD19	BUV737	1D3	BD	200
Siglec F	PE, BV421	E50-2440	BD	200
CD11C	PE-cy7, APC	N418	BioLegend	200
CD80	FITC	16-10A1	BioLegend	600
CD86	BV605	GL-1	BioLegend	200
PDCA1	BV650	927	BioLegend	100
PD-L1	BV785	10F.9G2	BioLegend	200
CD24	BUV396	M1/69	BD	400
CD103	PE	2E7	BioLegend	800
